# Electrophysiological Activity from the Eye Muscles, Cerebellum and Cerebrum During Reflexive (Classical Pavlovian) Versus Voluntary (Ivanov-Smolensky) Eye-Blink Conditioning

**DOI:** 10.1007/s12311-023-01613-6

**Published:** 2023-10-16

**Authors:** Neil P. M. Todd, Sendhil Govender, Peter E. Keller, James G. Colebatch

**Affiliations:** 1 UNSW Clinical School, Randwick Campus, Sydney, NSW 2052 Australia; 2 Department of Psychology, University of Exeter, Exeter, EX4 4QC UK; 3 Neuroscience Research Australia, UNSW, Sydney, NSW 2052 Australia; 4 MARCS Institute for Brain, Behaviour and Development, Western Sydney University Penrith, Kingswood, NSW 2751 Australia; 5 Center for Music in the Brain, Department of Clinical Medicine, Aarhus University, 8000 Aarhus, Denmark

**Keywords:** Cerebellum, Electrocerebellogram, Oculo-motor, Timing, Conditioned reflex, Ivanov-Smolensky conditioning

## Abstract

We report an experiment to investigate the role of the cerebellum and cerebrum in motor learning of timed movements. Eleven healthy human subjects were recruited to perform two experiments, the first was a classical eye-blink conditioning procedure with an auditory tone as conditional stimulus (CS) and vestibular unconditional stimulus (US) in the form of a double head-tap. In the second experiment, subjects were asked to blink voluntarily in synchrony with the double head-tap US preceded by a CS, a form of Ivanov-Smolensky conditioning in which a command or instruction is associated with the US. Electrophysiological recordings were made of extra-ocular EMG and EOG at infra-ocular sites (IO1/2), EEG from over the frontal eye fields (C3’/C4’) and from over the posterior fossa over the cerebellum for the electrocerebellogram (ECeG). The behavioural outcomes of the experiments showed weak reflexive conditioning for the first experiment despite the double tap but robust, well-synchronised voluntary conditioning for the second. Voluntary conditioned blinks were larger than the reflex ones. For the voluntary conditioning experiment, a contingent negative variation (CNV) was also present in the EEG leads prior to movement, and modulation of the high-frequency EEG occurred during movement. US-related cerebellar activity was prominent in the high-frequency ECeG for both experiments, while conditioned response-related cerebellar activity was additionally present in the voluntary conditioning experiment. These results demonstrate a role for the cerebellum in voluntary (Ivanov-Smolensky) as well as in reflexive (classical Pavlovian) conditioning.

## Introduction

The cerebellum has been considered to be difficult to record from non-invasively [1]. Reasons for this include the particular anatomy of the cerebellum causing electrical cancellation. Nevertheless, Andersen et al. [1] felt that recording cerebellar activity should be possible. Our prior observations have suggested that evoked potentials can be recorded from surface electrodes over the cerebellum in response to vestibular stimuli evoked using acoustic and inertial stimuli [2–5] and localised to the posterior cerebellum [6–9]. It was also found that cerebellar evoked potentials (CEPs) could be recorded to axial stimuli, which activate muscle afferents [9, 10]. These observations suggest that the ability to detect effects may depend upon the type of afferent stimulated whereby vestibular and axial projections can be detected, presumably reflecting the location of their cerebellar targets. A further important development was the realisation that, in addition to CEPs, the spontaneous activity from the cerebellum could also be detected using surface recordings (the electrocerebellogram or ECeG; [11, 12]). The ECeG has a characteristic high-frequency power spectral profile, including frequencies well above the high gamma range, extending up to several hundred Hz [11, 12]. CEPs of both vestibular and axial origin were associated with powerful short-latency modulation of the ECeG in the form of post-stimulus pausing and bursting [9]. These properties led to the proposal that the CEPs might be of climbing-fibre (CF) origin as post-stimulus pausing of simple spike activity is a known characteristic of CF responses (CFRs) [13]. Other properties of CEPs, their polarity, latency and laterality are also consistent with a climbing-fibre origin [5, 14].

In Todd et al. [15], we reported an eye-blink conditioning experiment using a mastoid tap unconditional stimulus (US). This is known to activate vestibular receptors and produce an eye-blink unconditioned response (UR) and was paired with auditory and visual conditional stimuli (CS). The experiment did produce eye-blink conditioning, albeit weak, which subsequently showed extinction. However, of particular interest were the observations showing ocular EMG activity and both unconditioned and conditioned post-CFR pausing in the ECeG, in turn, believed to be the substrate for the eye-blink conditioned response (CR) [16, 17]. These observations were confirmed in a subsequent eye-blink conditioning study using a more conventional trigeminal nerve US [18]. In particular, the presence of conditioned pausing in the high-frequency ECeG was observed to be aligned with the overt CR. Together, these support the interpretation of the modulation of the high-frequency ECeG as a manifestation of underlying simple spike activity. However, the results also supported the view that while conditioned cerebellar pausing may be necessary, other structures are likely involved in humans, including the basal ganglia [19]. A robust conditioned contingent negative variation (CNV) of likely central origin was also present prior to CR onset in parallel with the changes in the ECeG.

The present experiment is an extension of the Todd et al. [15] design in which the single-tap US has been replaced by a double-tap US. In addition, this classical eye blink conditioning procedure was followed by an experiment in which subjects were instructed to blink in synchrony with the double head taps, thus allowing direct comparison of cerebellar activity during reflex and voluntary blinks. The latter procedure is known as Ivanov-Smolensky conditioning (ISC) in which subjects are presented with a conventional CS followed by either a command US or an imperative US which signals a command [20], here a command to blink. The unconditioned responses (URs) thus include both an instructed voluntary unconditioned blink UR (v-UR) and a reflexive unconditioned blink UR (r-UR). Several authors have reported successful ISC in which subjects learn a voluntary CR (v-CR) which anticipates the US [20], and prior work has confirmed that human subjects can learn timed voluntary blinks which mimic classical conditioning [21, 22]. It may be that mechanisms of voluntary and reflexive motor learning overlap [23] and that Purkinje cells participate in learning or predicting sequences of responses in both cases [24]. Indeed, it has been suggested that ISC and classical (Pavlovian) conditioning may be one and the same, not least because the temporal context for the establishment of v-CRs, i.e. a short (a few 100 ms) CS-US inter-stimulus interval, appears to be the same as for reflexive CRs (r-CRs) [20, 25]. If sensorimotor synchronisation is viewed as a form of conditioning, then the cerebellum might play a role in both learning and timing. Thus, this study could provide evidence for the role of the cerebellum in sensorimotor synchronisation, consistent with the supposed role of the cerebellum in motor timing [26, 27]. The hypothesised link between conditioning and timing might be manifest in activity in specific frequency bands. High-frequency bands are of particular interest to the extent that they support sensory-motor integration by allowing both rapid local processing and the synchronisation of nodes within functional networks distributed across the cerebellum and cerebrum [28, 29]. A further aim was thus to look for evidence for movement related to high-frequency EEG in parallel with that in the ECeG.

## Methods

### Participants

A total of 11 healthy adults (five female and six male) without prior history of vestibular, hearing or neurological impairment were recruited from the general community and staff and students at the Prince of Wales Hospital, the University of New South Wales and Western Sydney University. Written informed consent was obtained prior to testing in accordance with the Declaration of Helsinki. The study was approved by the local ethics committee (South Eastern Sydney Local Health District Human Research Ethics Committee).

### Unconditional and Conditional Stimuli

The impulsive (tap) unconditional stimulus (US) consisted of a double presentation, with 600 ms inter-tap interval, of a 3rd-order gamma waveform with a 4 ms rise time, applied to the left mastoid. This is an effective means of activating the utricle [30] and also for evoking CEPs [14]. Customised software was used to generate the gamma waveform using a CED Power1401 and fed to a power amplifier (model 2718, Brüel & Kjaer P/L, Denmark). A hand-held mini-shaker (model 4810, Brüel & Kjaer P/L, Denmark) with an attached perspex rod was used by a single experimenter (S.G.) to deliver the stimulus. The intensity used was 20 V peak, equivalent to approximately 14 N peak force level (FL) and delivered using a positive phase polarity (i.e. initial movement of the perspex rod towards the head). This results in an approximately incompressive lateral low-frequency acceleration of the whole head without significant higher frequency bone conduction [2, 3].

The preceding auditory conditional stimulus (CS) consisted of a 1200 ms, 2 kHz sinusoidal tone starting 600 ms immediately prior to the first mastoid stimulus and ending at the same time as the second mastoid tap. The combined CS+US thus included three overt events at 600-ms intervals, CS onset, first US onset and second US onset, implying a fourth event at 1800 ms, referred to as an implied (silent) 3rd US onset. Tones were generated using Signal software (version 6.02, Cambridge Electronic Design, Cambridge, UK) and a CED Power1401 interface, fed to a customised amplifier and delivered bilaterally using insert earphones. The stimulus intensity used was − 60 dB re 5 volts peak (equivalent to approximately 80 dB peak sound pressure level (pSPL) or about 77 dBA SPL).

### EMG/EOG and EEG/ECeG Recordings

**Fig. 1:**
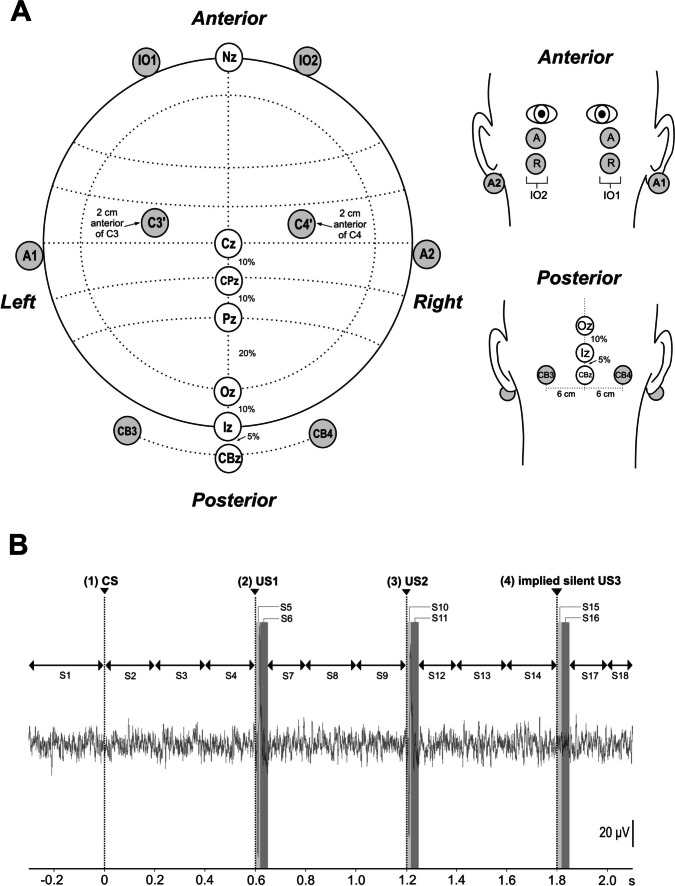
**A** Axial and coronal views of the experimental montage (grey electrodes) consisting of infra-ocular (IO1, IO2), central (C3’, C4’) and cerebellar (CB3, CB4) electrodes used to record EOG/EMG, EEG and ECeG, respectively. The infra-ocular montage consisted of active (A) and reference (R) electrodes positioned slightly lateral and beneath the eyes. EEG recordings from over frontal eye fields were made 2 cm anterior to the conventional C3 and C4 locations. ECeG recordings were made lateral (6 cm) to the midline CBz location, which is situated 5% below the Iz level. EEG and ECeG recordings were referenced to linked earlobes (A1, A2). **B** The recording epoch demonstrating the stimulus onsets (CS, US1, US2, an implied third stimulus US3) and segmentation used for analysis (refer to Table 1 for abbreviations). The single subject data shown is taken from the CB4 electrode. Note differing durations of the segments, shorter around the time of the expected response to the US

Simultaneous measurements were made from beneath the left and right eyes (infra-ocular, consisting of both EMG and EOG: IO1 and IO2 referenced to electrodes 2 cm below), the presumed frontal eye fields (EEG: C3’ and C4’, 2 cm anterior to C3 and C4) and over the posterior fossa (ECeG: CB3 and CB4), referenced to linked ear lobes, using Ag/AgCl electrodes (Fig. 1A). The posterior fossa cerebellar electrodes were 6 cm lateral from the CBz location, itself 5% below Iz. The earthing electrode was placed over the sternoclavicular joint. The total length of the recording epoch was 2.1 s with a 300-ms interval preceding the onset of the auditory stimulus and the impulsive mastoid stimuli given 600 ms and 1200 ms later. Signals were amplified (EMG/EOG: × 10 000; EEG and ECeG: × 20,000), filtered (5 Hz–1 kHz) and sampled (10 kHz) using a CED Power1401 and recorded using Signal software (Cambridge Electronic Devices, Cambridge, U.K.). Subjects were positioned in a recumbent or semi-recumbent position during recordings with their heads resting on a pillow, to minimise any potential contamination from the neck muscles.

### Experimental Procedure

**Fig. 2:**
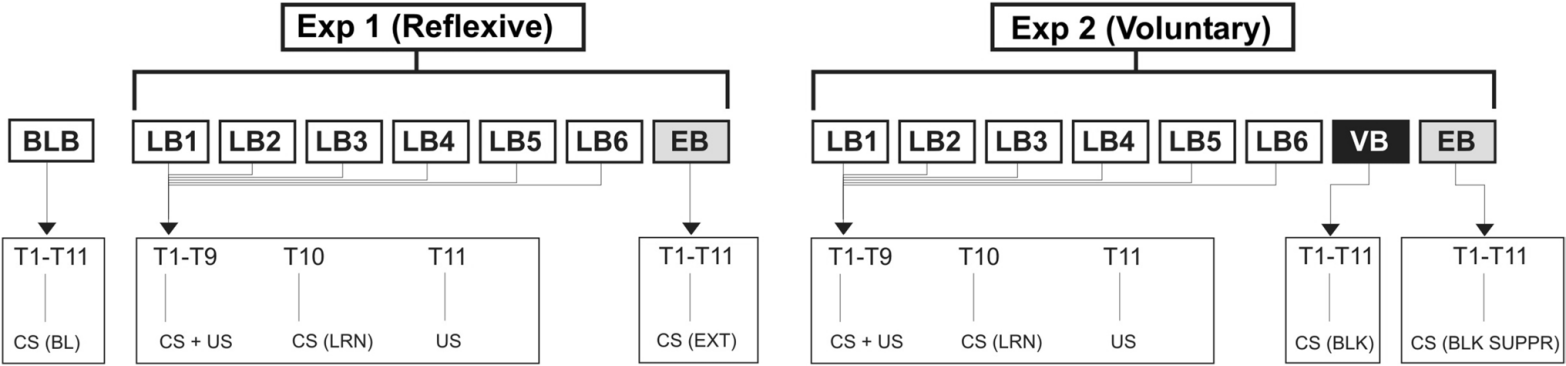
The sequence for experiments 1 (reflexive) and 2 (voluntary) including the preceding baseline block (BLB) of eleven CS alone trials (CS (BL)). For both experiments, six learning blocks were carried out (LB1-6) with each consisting of eleven trials (T1–T9: CS+US trials, T10: CS alone (CS (LRN)) and T11: US alone). For experiment one, an extinction block (EB; grey box) was recorded after learning and consisted of eleven CS alone trials (CS (EXT)). For experiment two, two blocks were recorded after learning. A voluntary blink block (VB, black box) in which subjects were instructed to blink when the US would have occurred (CS (BLK)) and an equivalent extinction block to experiment one in which subjects were instructed to not blink (CS (BLK SUPPR))

For experiment 1 (reflexive conditioning), we used a procedure based upon an adaptation of that of Teo et al. [31] (Fig. 2). They used electrical stimulation of the supra-orbital nerve as an eye-blink unconditional stimulus (US), combined with an auditory tone conditional stimulus (CS). The present experiment substituted the double vestibular mastoid tap for the supra-orbital nerve stimulus and like Teo et al. [31], consisted of six learning blocks (LB) of 11 trials, with an inter-trial interval of 8–12 s. The whole experiment was preceded by a ‘baseline’ block (BLB) of eleven CS alone trials (CS (BL). In the following 6 learning blocks, the first nine trials always consisted of an auditory CS plus the US, while the 10th trial was the CS alone (CS (LRN)) and the 11th trial was the mastoid tap US alone. This was followed by a seventh ‘extinction’ block (EB) of eleven CS alone trials (CS (EXT)).

For experiment 2 (voluntary eye blinks), the stimulus and block procedure were identical to that of experiment 1 except that, on the six learning blocks, the subjects were given explicit instructions to blink in time with the head taps and this was demonstrated. For the seventh block of CS alone trials, which in the first experiment was the extinction block, the subjects were further instructed to voluntarily blink (VB) at the time when the taps would have occurred (CS (BLK). Then, on a final block of CS alone trials, subjects were instructed to not blink (CS (BLK SUPPR), equivalent to the extinction block (EB).

### Statistical Analysis

For each subject, automated measurements were made using a MATLAB script on a trial-by-trial basis of the baseline corrected RMS total power of EMG/EOG in the infra-ocular leads (IO1/IO2), of EEG in the central leads (C3’/C4’) and of ECeG in the cerebellar (CB3/CB4) leads. Due to the large individual variability in response amplitudes, analyses were performed on log-transformed data. A single trial epoch was segmented into 18 segments of unequal durations, as given in Table 1 and Fig. 1B. Segments 5, 6, 10, 11, 15 and 16, at the times of expected responses, were only 20 or 30 ms long and shorter than the remainder. Means across the 8 blocks for the two experiments were made for the five trial types: for experiment 1, CS alone during baseline (BLB, T1-T11), CS+US during learning (LB1-6, T1-9), CS alone during learning (LB1-6, T10), US alone during learning (LB1-6, T11) and CS alone during the extinction block (EB, T1-11, CS (EXT)); for experiment 2, CS+US during learning (LB1-6, T1-9), CS alone during learning (LB1-6, T10), US alone during learning (LB1-6, T11), CS alone during voluntary blinks (VB, T1-11) and CS alone during the voluntary ‘extinction’ block (EB, T1-11). These were further averaged across subjects to make grand means.

**Table 1:** Trial segmentation of RMS power for statistical analysis

Segment	Interval	Name	Description
S1	300 ms	BL	Baseline
CS onset (CS–time point 1)–0 ms			
S2	200 ms	AS	The initial (‘alpha’) part of the CS
S3	200 ms	CS1	The interval which followed the CS
S4	200 ms	Pre-CFR1	Immediately prior to the 1st tap
1st tap onset (US1–time point 2)–600 ms			
S5	0–20 ms	CFR1	The 1st CFR period post-US1
S6	20–50 ms	Post-CFRP1	The post-CFR pause post-US1
S7	50–200 ms	Post-CFRB1	The post-CFR burst post-US1
S8	200 ms	CS2	The CS interval which followed the 1st US
S9	200 ms	Pre-CFR2	Immediately prior to the 2nd tap
2nd tap onset (US2–time point 3)–1200 ms			
S10	0–20 ms	CFR2	The 2nd CFR period post-US2
S11	20–50 ms	Post-CFRP2	The post-CFR pause post-US2
S12	50–200 ms	Post-CFRB2	The post-CFR burst post-US2
S13	200 ms	CS3	The CS interval which followed the 2nd US
S14	200 ms	Pre-CFR3	Immediately prior to an implied (silent) 3rd tap
Implied (silent) tap onset (US3–time point 4)–1800 ms			
S15	0–20 ms	CFR3	The 3rd implied CFR period post-US3
S16	20–50 ms	Post-CFRP3	The post-CFR pause post-US3
S17	50–200 ms	Post-CFRB3	The post-CFR burst post-US3
S18	100 ms	Return	Return to baseline

Definition of segments with the main events they included. Note differing durations, with short durations following the times of the US to capture the evoked CFR. CFR, climbing fibre response

As there was an unequal number of trials allocated to each of the five types, the analysis of variance (ANOVA) was conducted in two stages. An initial repeated measures ANOVA using ‘block’ (1–6), ‘trial-type’, ‘side’ and ‘segment’ was performed to test for a block effect and then further concatenated across blocks. The responses during the five trial types could then be compared by ANOVA using within-subjects factors of ‘trial-type’ (CS+US, CS alone during learning, US alone during learning and CS alone during extinction), ‘side’ and ‘segment’. To test for any conditioning effects in the CB leads, the ANOVA was repeated for CS alone trials only. To compare across experiments, three common trial types were extracted (CS+US, CS alone during learning, US alone during learning) and combined with a within-subjects factor of ‘experiment’.

The CS + US trial type most clearly exhibited the synchronised blinks and correlated cerebellar activity, features of particular importance for this report. In the description that follows, we have assumed that short latency events following the tap stimuli were reflex or reflexive. Events which preceded (i.e. anticipated) the stimuli, occurring when the subjects were asked to respond and when they could predict the timing of the stimuli, were considered conditioned or voluntary.

### Spectral Power Analysis

After recording EMG/EEG/ECeG, we performed spectral power analyses of the six channels (IO1/2, C3’/C4’, CB3/4) using the continuous wavelet transform as implemented in the MATLAB toolbox (R2019b, MathWorks, Natick, CA). In the present analysis, a Morlet wavelet was employed at a density of 24 voices per octave over 9 octaves. The CWTs were further transformed to scaleograms (time-frequency images) from the absolute value of the CWT and rescaled to be in dB per Hz re 1 μV^2^. Scaleograms were computed for all trials and then further split into eight frequency bands; delta (*δ*: 1.8 Hz–4 Hz), theta (*θ*: 4–7.8 Hz), alpha (*α*: 7.8–12.5 Hz), beta (*β*: 13–30 Hz), gamma (*γ*: 30–80 Hz), ultra-gamma (u-*γ*: 80–160 Hz), very high frequency (VHF: 160–320 Hz) and ultra-high frequency (UHF: 320–640 kHz). These were then further segmented using the same time boundaries as for the RMS analyses and submitted to ANOVA. Wavelet coherence was also computed using the same MATLAB toolbox.

## Results

### Grand Means and RMS Power ANOVA

**Fig. 3:**
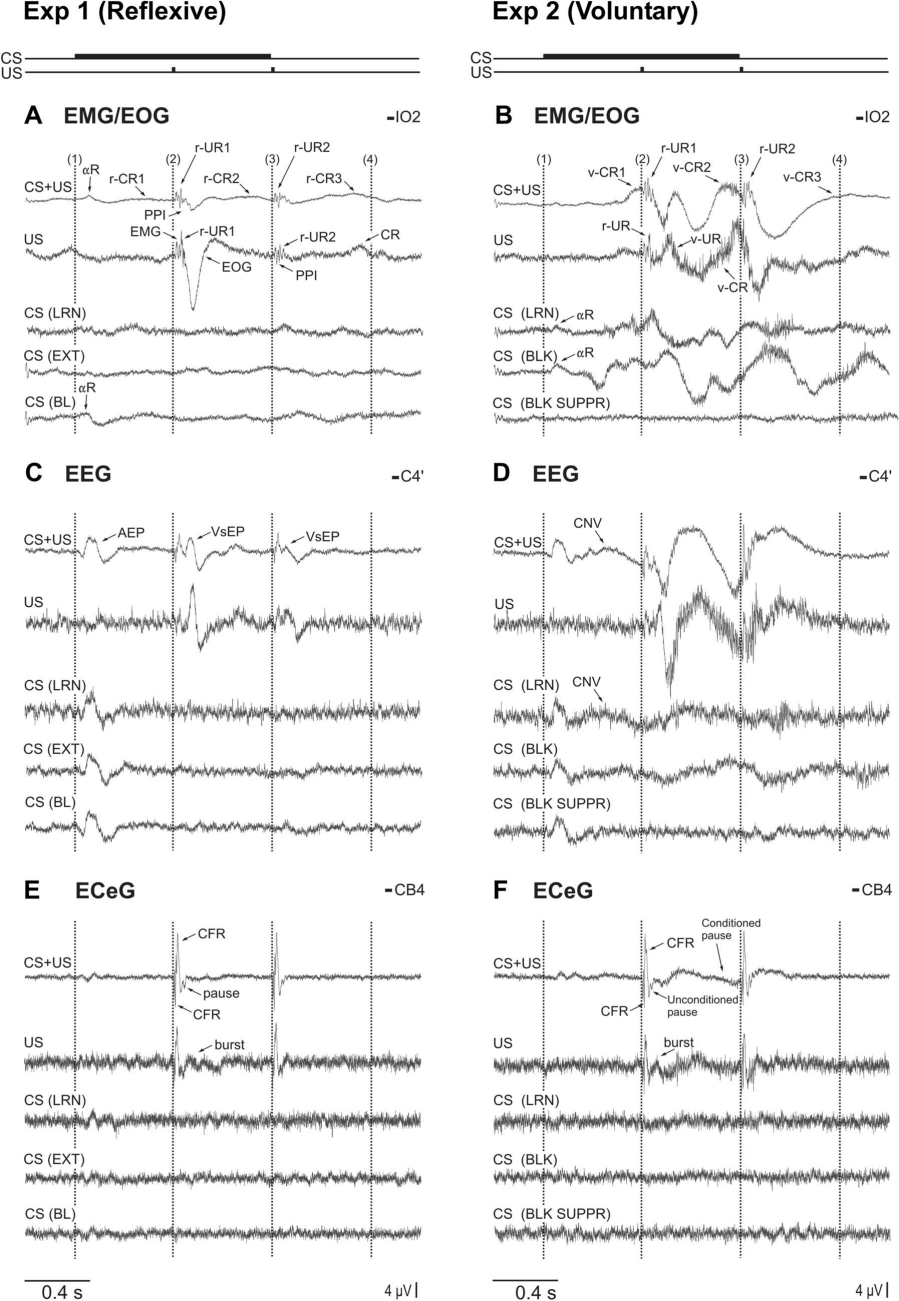
Grand unrectified means for the right-sided infra-ocular (**A**, **B**; IO2), central (**C**, **D**; C4’) and cerebellar electrodes (**E**, **F**; CB4) for each of the five trial types for experiment 1 (reflexive conditioning; left column) and experiment 2 (voluntary timed eye blinks; right column). For experiment 1, the infra-ocular electrodes show large EMG/EOG potentials (blink UR) which are diminished upon the second US stimulus in US alone and CS+US trial types. Central electrodes show auditory evoked potentials (AEP) in all CS onset trial types with vestibular evoked potentials (VsEPs) after the US taps. Cerebellar electrodes show a presumed climbing-fibre response (CFR) following both US onsets and subsequent pause-burst activity. For experiment 2, the infra-ocular electrodes demonstrated large timed blink responses which often peaked close to the US onsets whereas voluntary blinks during CS alone trials were infrequent and peaked later. For the central electrodes, auditory evoked potentials (AEP) were present in all CS onset trial types, similar to experiment 1. Pre-movement potentials (CNVs) were present in the CS+US and CS alone during learning trial types. Findings for the cerebellar electrodes were similar to that for reflexive responses. LRN, learning; EXT, extinction; BL, baseline; BLK, blink; BLK SUPPR, blink suppression; CNV, contingent negative variation; CS, conditioned stimulus; US, unconditioned stimulus; PPI, pre-pulse inhibition; CR, conditioned response; UR, unconditioned response; r-, reflexive; v-, voluntary

ANOVA showed no significant block effect indicating consistent behaviour across the two experiments and justifying averaging over blocks. Figure 3 shows grand means of unrectified signals for three right-sided channels (the infra-ocular IO2 for EMG/EOG, the central C4’ for EEG and the cerebellar CB4 for ECeG) and five trial types for experiments 1 and 2, respectively. The findings for the equivalent channels on the left were nearly identical and therefore are not illustrated. Equidistant time points (1–4) were marked every 600 ms corresponding to onsets of the CS (1), US1 (2), US2 (3) and an implied (silent) US3 (4). The CS alone baseline condition was presented as the fifth trial type.

Considering first the periocular responses, in experiment 1, a large EMG/EOG signal, corresponding to the blink r-UR1, was produced in IO channels (Fig. 3A) in response to the first tap of the US alone trial type (time point 2). When preceded by a CS, however, the r-UR to the same tap was much reduced, corresponding to pre-pulse inhibition (PPI), as was the r-UR to the 2nd tap in both CS+US and US-only trials (time point 3). The CRs, by definition, occurred prior to the onset of the UR, and thus, the smaller URs due to PPI are not relevant. For the CS+US trials, weak r-CRs were present prior to the US taps at IO2. Weak r-CRs can also be seen at the time of the (implied) third stimulus (CR3 and CR, part A). Larger, robust double-blink responses were present in experiment 2. Starting with the response to the US-only trials (Fig. 3B), this began in a manner similar to that of experiment 1, with a v-UR1 but was followed by a second blink which reached a peak close to the onset of US2 (time point 3) and thus a form of v-CR. For the CS+US trials, the first voluntary CR (v-CR1) began before US1 and reached a peak close to the onset of US1 and the associated reflexive EMG. The second conditioned voluntary blink (v-CR2) also started in advance of the stimulus, with voluntary EMG occurring ahead of the associated stimulus-evoked EMG. In addition, the timing of the second response was more accurate with the US than when subjects were asked to blink to the CS alone (CS BLK). A weak third voluntary blink (v-CR3) associated with the implied US3 was also evident. A more robust v-CR3 was present in CS (BLK) trials. These effects were reflected in the ANOVA of the RMS IO data (Table 2) where a main effect of ‘trial-type’ was obtained for experiment 2 but not experiment 1.

**Table 2:** ANOVA of ECeG RMS power

Electrode	All trial types	*df*	EXP 1	*p*	EXP 2	*p*
	Factor		*F*		*F*	
CBs	TT	4,40	2.5	ns	5.8	< 0.01
	SIDE	1,10		ns		ns
	SEG	17,170	11.6	= 0.001	8.6	< 0.001
	TT*SEG	68,680	12.1	< 0.001	11.1	< 0.01
	CS-only trial types		EXP 1		EXP 2	
CBs	TT	2,20	0.8	ns	7.5	< 0.01
	SIDE	1,10		ns		ns
	SEG	17,170	2.4	< 0.05	1.8	ns
	TT*SEG	34,340	1.7	ns	1.4	nsI

TT, trial type; SEG, segment

In the central EEG channels, auditory evoked potentials (AEP) were present for trials with a CS for both protocols (C4’, Fig. 3C, D). For experiment 1, presumed vestibular evoked responses (VsEPs) were present for the US and CS+US trials. For the voluntary task, the earliest response in the US trials resembled the reflexive response, but a large positivity followed. In the CS+US trials, a small negative excursion and presumed contingent negative variation (CNV) started prior to the onset of v-CR1.

For the ECeG in experiment 1 (Fig. 3E), each of the mastoid taps was followed by large CEPs with an initial positivity. As we have previously reported [14], the latency on the side contralateral of the stimulus was shorter than ipsilaterally (overall means; contra: 11.4 ms, vs. ipsi: 13.7 ms, *p* < 0.001). The CEP was followed by a pause and small burst in the ECeG (Fig. 3E, top 2 traces). For experiment 2 (Fig. 3F), similar features were present, with the initial positivity having latencies of 11.6 ms and 13.5 ms for the contralateral and ipsilateral sides, respectively. There was no significant effect of experiment on the latencies (*p* > 0.05). The initial CEP amplitudes were nearly identical for both ipsilateral and contralateral responses, for both experiments. There was an additional feature of following slow negativity, clearest for the CS+US case.

**Fig. 4:**
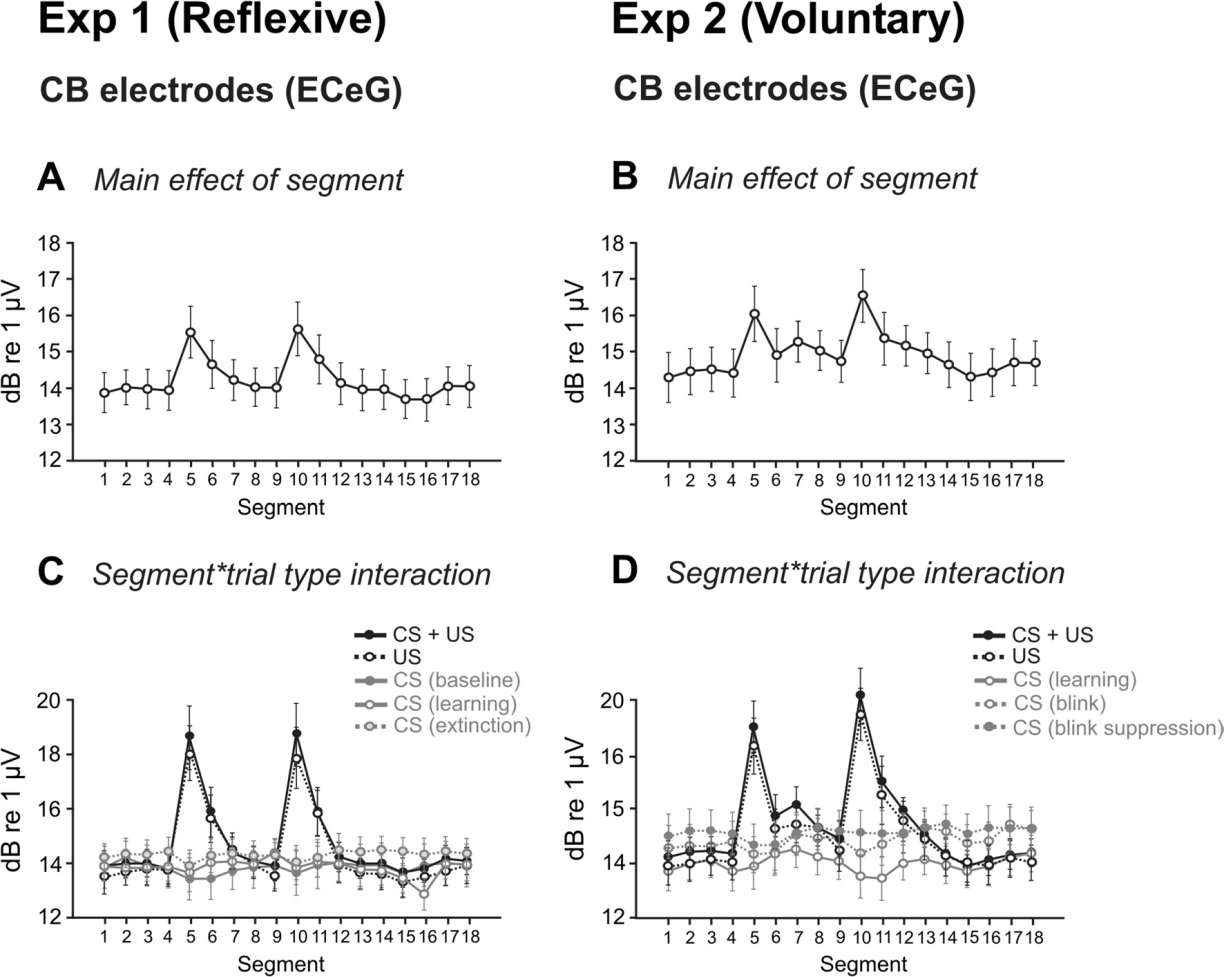
Marginal means using RMS averaging of sequential segments, for the effects of ‘segment’ and the interaction of ‘segment’ and ‘trial type’ cerebellar electrodes for experiment 1 (**A**, **C**; left column) and experiment 2 (**B**, **D**; right column). Overall, similar patterns were seen for both movement tasks. Note that the segment durations are not constant

The segmented ECeG RMS power and ANOVA were dominated by the two clear peaks occurring with the US stimuli (Table 2, Fig. 4A–D). The CS alone trials show some cyclical reduction in power around the time of the stimuli for both experiments manifest as a ‘segment’ effect (*p* < 0.05 for the reflexive case), including for the implied US3 (segment 15), and a main effect of ‘trial-type’ in experiment 2 (*p* < 0.01).

### Wavelet Power/Coherence

**Fig. 5:**
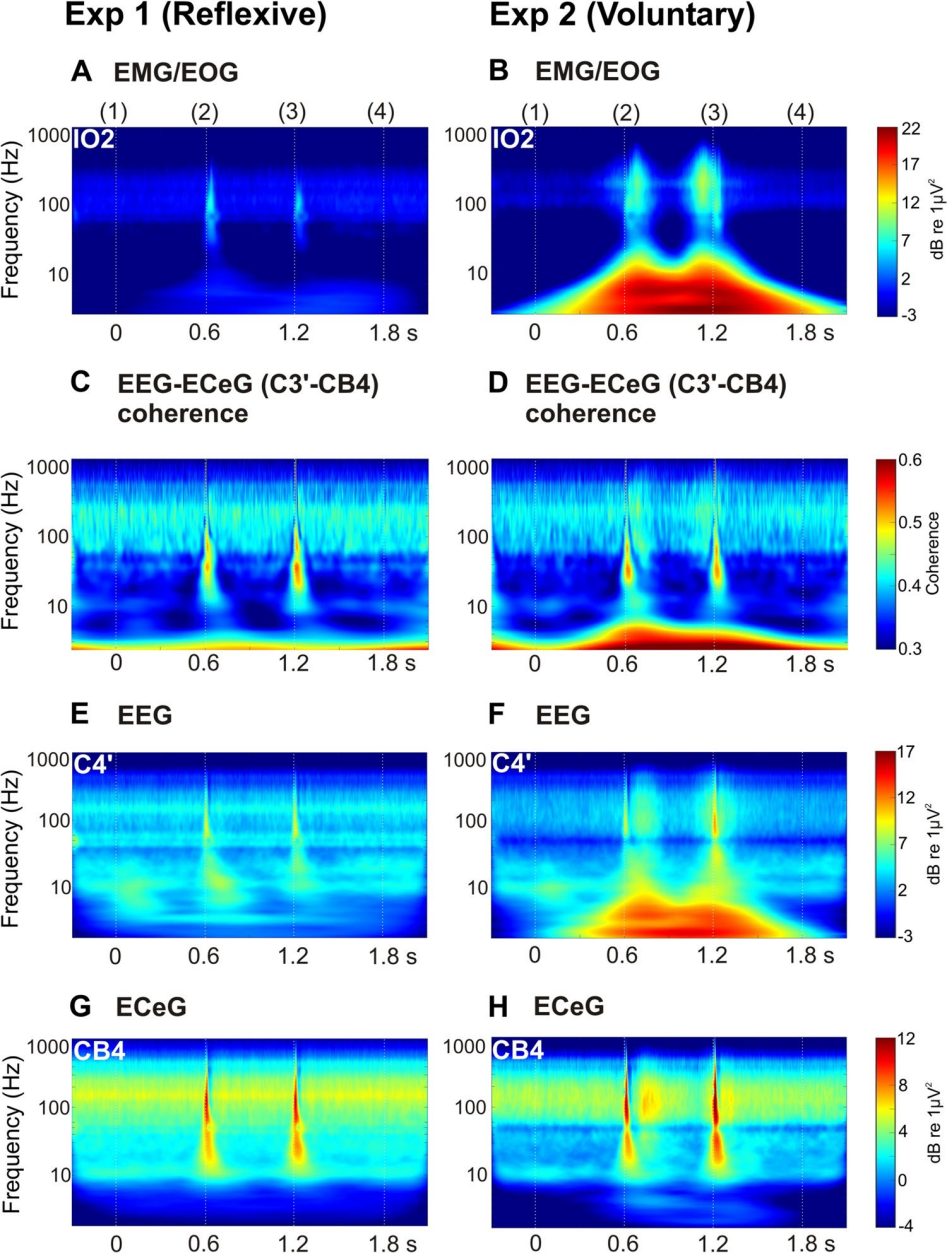
Scaleograms for the infra-ocular EMG/EOG (**A**, **B**; IO2), central EEG (**E**, **F**; C4’) and cerebellar ECeG (**G**, **H**; CB4) for experiment 1 (reflexive conditioning) and experiment 2 (voluntary conditioning) for the CS+US trial type. C3’-CB4 EEG-ECeG coherograms (**C**, **D**) are illustrated for comparison and were similar to the corresponding C4’-CB3 coherograms

**Fig. 6:**
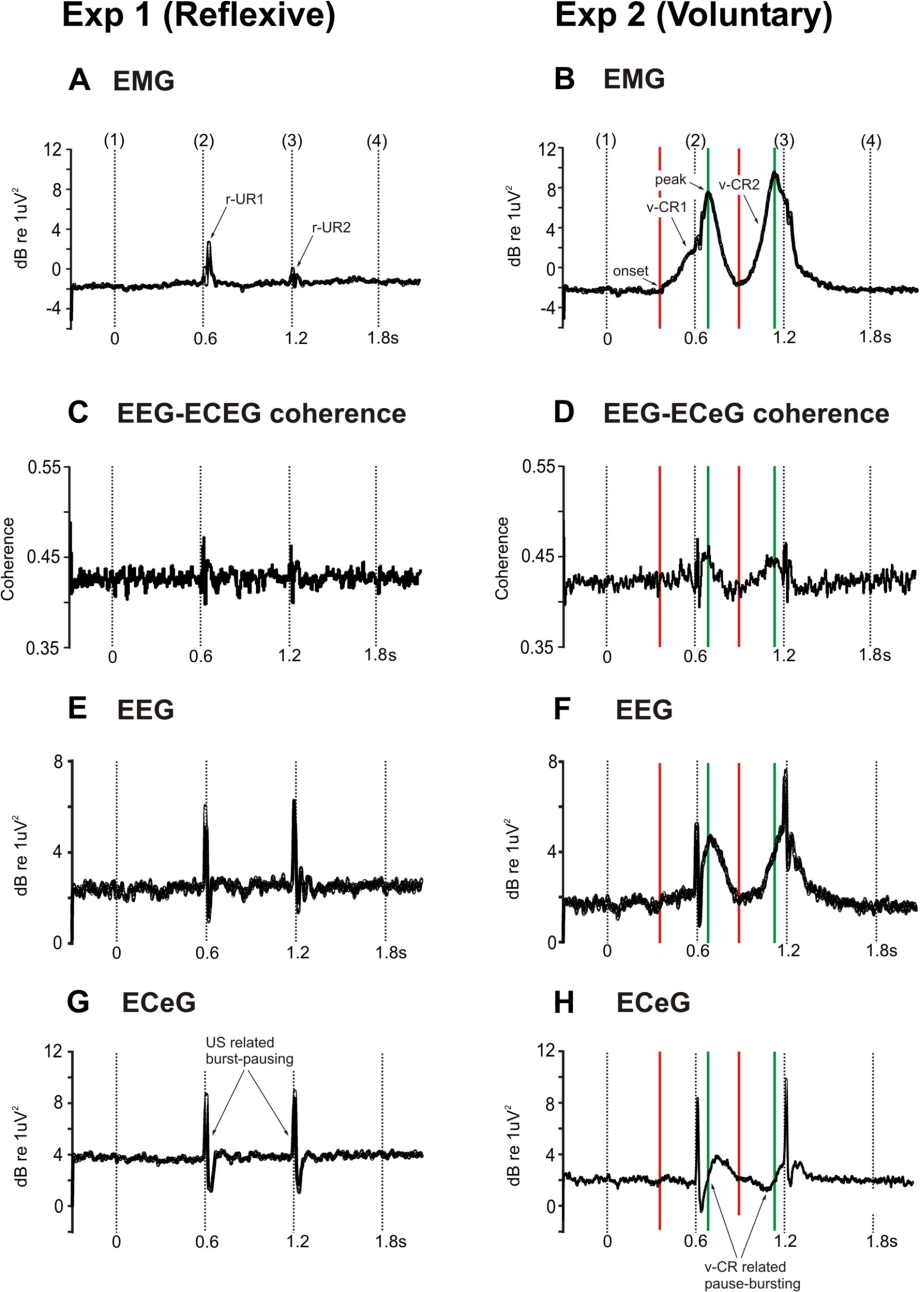
VHF power for the infra-ocular (**A**, **B**), central (**E**, **F**) and cerebellar electrodes (**G**, **H**) and CS+US trial types for experiment 1 (reflexive conditioning) and experiment 2 (voluntary conditioning). VHF power shown reflects the averages between the right and left sides for EMG/EOG, EEG and ECeG graphs. VHF EEG-ECeG coherence bands are also illustrated for comparison (**C**, **D**) and are the average coherence of C3’-CB4 and C4’-CB3 comparisons. The CFR peaks (6G-H) relate to the USs and not the CRs. v-CR-related pause-bursting is present in experiment 2 (6H). Red lines reflect the onset of the v-CRs, whereas green lines reflect the peak EMG/EOG CR

Figure 5 shows scaleograms, and Fig. 6 the associated VHF (160–320 Hz) power. The C3’-CB4 coherograms (Fig. 5C, D) and VHF mean C3’-CB4/C4’-CB3 coherence (Fig. 6C, D) are included. The CS+US trial type is illustrated as this showed the clearest effects. In the IO channel, the high-frequency EMG and low-frequency EOG signals are segregated, most clearly for experiment 2 (Fig. 5). Whereas the EOG cuts off between 10 and 20 Hz, the EMG picks up between 40 and 50 Hz and spreads upwards to several hundred Hz (Fig. 5B). The EMG also helps clarify the timing of the blinks for experiment 2, where the first of the pair peaks occurred after the first US tap, but where the second is clearly anticipating the 2nd US tap, given at 1.2 s (3). The second EMG burst is also larger by about 2 dB, consistent with more accurate (less variable) timing.

High-frequency activity was present in the EEG (Figs. 5 and 6E, F), including in the spontaneous EEG, up to several hundred Hz. Of note was the presence of high-frequency movement-related EEG activity in parallel with, but slightly lagging, the ocular EMG (part 6B) for the voluntary case. The prominent peaks in the ECeG were related to the vestibular US in both experiments. However, in the voluntary case, some additional v-CR-related modulation in the form of conditioned pause-bursting was present within the high-frequency component of the ECeG (Figs. 5 and 6 G versus H). This was manifested in the combined high-frequency power ANOVA across the two tasks with the common three trial types (CS+US, CS learning, US alone) which showed a significant three-way interaction of ‘experiment’ by ‘trial-type’ by ‘segment’ for the u-*γ* band (*F*(34,340) = 2.9, *p* < 0.05) and a trend to significance for the VHF band (*F*(34,340) = 2.4, *p* = 0.068). As with the EEG, the ECeG burst appeared to lag the EMG activity. Thus, the pause phase occurred during the rising phase of the ocular EMG, while the subsequent burst peaked during the falling EMG. There was also evidence of US-related EEG-ECeG coherence for both tasks, manifest as interactions of ‘trial-type’ by ‘segment’ in the *β*, *γ* and u-*γ* bands (respectively, *p* < 0.005, *p* = 0.001 and *p* = 0.055 for the reflexive case and *p* < 0.005, *p* = 0.001 and *p* < 0.05 for the voluntary case). For the voluntary task, there appeared to be possible evidence of v-CR-related high-frequency EEG-ECeG coherence around the time of the two stimuli which seemed to follow the EMG (Figs. 5 and 6C, D), but this did not reach statistical significance.

## Discussion

### Changes in the ECeG

We have used electrodes placed over the posterior fossa to record evoked responses and electrical activity (ECeG) that we believe arises from the cerebellum. Samuelsson et al. [32] calculated that ECeG should be detectable in surface recordings but weaker than cerebral EEG. Others have been able to make recordings of cerebellar activity using surface recordings. For example, Bosch et al. [33] confirmed that recordings from over the cerebellum were distinct from those at Oz. In our present study, we observed systematic changes in the ECeG which provide evidence of the participation of the cerebellum in conditioning. In the two experiments, we compared the electrophysiological features associated with four types of responses—both voluntarily and reflexive URs to a US alone (r-URs vs. v-URs) and both voluntary and reflexive CRs (r-CRs vs. v-CRs) which anticipate the US, given a prior CS. The nature of the four types of response was distinct, and the first and the second of the pair of each type differed for both experiments. In experiment 1 for US-only trials, both r-UR EMG/EOG responses followed the US, with the second being smaller than the first, consistent with pre-pulse inhibition (PPI) [34]. In experiment 2, for the US-only trials, the r-UR EMG was still present on the first US tap, but this was followed by a v-UR and then a larger anticipatory v-CR before the second US tap. Despite these clear differences between the two experiments, ECeG changes were virtually identical around the time of both the US pairs, suggesting that they were dominated by afferent input. For the CS+US trials in both experiments, CRs were present, although only weakly for experiment 1, and in experiment 2, the second of the v-CRs clearly anticipated the second of the US pair. However, in addition to the US-related ECeG changes, we also observed superimposed pause-bursting correlated with the v-CRs, analogous to conditioned pausing in classical conditioning in the circumstance that robust conditioning occurs, as observed in Todd et al. [18] and first hypothesised by Albus [16]. Thus, the Albus model of cerebellar learning may generalise to voluntary conditioning.

The ECeG changes were also accompanied by the presence of a CNV in central leads prior to the onset of voluntary movement and modulation of the high-frequency EEG. A CNV was not apparent in experiment 1. We have previously reported that this form of US produced only weak conditioning, which rapidly habituates [15]. In contrast, a stronger trigeminal nerve US produced more robust conditioning [18], along with conditioned pausing in the ECeG and a CNV. When subjects determine the timing of their blinks, a readiness potential is present prior to the movement, maximal over the vertex [35]. Where movements are contingent upon stimuli, a CNV may be present, for both forms of conditioning [36], as also observed in Todd et al. [18]. This is likely to be indicative of a role of the cerebrum, including basal ganglia, in addition to the cerebellum.

### The High-Frequency ECeG and the Oculomotor System

Given the presence here of pausing in the high-frequency ECeG, an important issue is the possible role of the oculomotor system in voluntary eye blinks, especially since Purkinje cell pause-bursting occurs with saccadic eye movements [37]. Reflex eye blinks are largely mediated at the brainstem level, e.g. [38]. The ocular-motor areas of the cerebellum include the flocculus/paraflocculus, the nodulus/ventral uvula, two areas shared with the vestibulo-ocular system, plus the oculomotor vermis (OMV) [39]. The OMV, located within lobules V–VII of the vermis, is situated relatively close to the skull surface near Iz and thus plausibly within reach of the window for non-invasive electrophysiology of the posterior cerebellum. This site has been used successfully for TMS influence of the saccadic system [40]. The OMV is known to be involved in saccadic eye movement and may participate in reflexive blink-related eye movements as well as voluntary blinks [39]. The observed high-frequency pausing behaviour in the ECeG is reminiscent of Purkinje cell pausing and bursting units in the saccadic system of the OMV. The timing of the v-CR-related ECeG pause-bursting we observed occurred during the rising and falling edges of the EMG and is thus consistent with a role for the cerebellum in sharpening movement by acceleration after onset and breaking to assist accurate targeting [38].

### High-Frequency Components of the EEG

In addition to the high-frequency spectral components in the ECeG, we also observed movement-related, high-frequency EEG along with EMG. Movement-related sensory-motor high-gamma frequency EEG has been described, most commonly in brain-computer interface (BCI) research using the electrocorticogram (ECoG) approach, and is believed to be related to somatosensory feedback, e.g. [41]. Our data are consistent with the interpretation that the high-frequency EEG is related to reafference but with the range extended upwards well beyond the ultra-gamma range [42]. Other BCI/ECoG studies have demonstrated that the presence of EMG-related very high frequencies in motor cortex [43] and high-frequency ECeG-EEG coherence here seemed related to EMG.

### Sensorimotor Synchronisation as a Form Conditioning

While only weak classical conditioning was evident in the present study, despite the use of dual stimuli, robust voluntary conditioning in the form of voluntary timed blinks was evidence of the presence of sensorimotor synchronisation. Establishing and maintaining synchrony with external pacing sequences are dissociable processes that are likely to rely on distinct mechanisms [44]. Much research on sensorimotor synchronisation has addressed the mechanisms that enable individuals to maintain coordination [45–47]. The process of establishing synchrony at the start of a trial has received less attention (in fact, the first few movements, typically finger taps, are usually not analysed in order to focus on the relatively stationary time series that follow). From previous work, we know that establishing synchrony at the start of a trial, or re-establishing it after an unexpected tempo change, typically takes around three cycles [44, 46, 48, 49]. This process of temporal adaptation relies on reactive error correction processes that implement compensatory adjustments to movement timing with onset latencies 100–250 ms (depending on tempo) in the movement cycle immediately following a timing perturbation in the pacing sequence [46]. Our findings provide detailed evidence of the events occurring both centrally and for the cerebellum at the onset of a sequence performed in two distinct ways.

Although conditioning, either classical or voluntary, and sensory-motor timing are traditionally distinct areas in cerebellar research, they can be seen to be related at multiple levels. At one level, conditioning and repetitive, timing-specific movement may rely on common cerebellar substrate for representing temporal information [50], while at another level, they can be viewed as different aspects of the same fundamental process, i.e. conditioning can be viewed as a form of adaptive motor timing, while adaptive motor timing can be viewed as a form of learning. The latter concept relies on a dual-route framework whereby a neural pathway involving the inferior olive and CFs mediates motor learning based on reflex conditioning, while a pathway that projects to cerebellar Purkinje cells via the pontine nuclei and mossy fibres mediates learning based on memory traces [51, 52].

Given the above view, adaptive timing to a vestibular metronome tap can be considered to be a form of learning where the stimulus serves as both an US (via the CF pathway) and a CS (via the mossy fibre pathway). There is a clear example of this in the US-only trials for experiment 2 where a v-CR appears in anticipation of the 2nd US tap, as if the first US tap had acted as a CS. It should be noted though that, unlike with the continuous auditory CS, which overlaps with the double tap US and hence is a form of delay conditioning, the conditioning which occurs as a result of the double tap is a form of trace conditioning. In this form of conditioning, a memory trace of the empty inter-stimulus interval is required and is thought to involve the hippocampus as well as cerebellum [53]. The hippocampus is also known to be involved in the memory for voluntary movement [54].

The doubling up of a vestibular stimulus as both CS and US can be generalised to a sequence of taps, where each successive stimulus may trigger both an UR to the current and a CR timed for the following tap. In our present study, any classical conditioning was weak, consistent with the blinks remaining primarily URs to the vestibular US for the reflex task. For the ISC conditioning, the timing of the second v-CR appeared improved by the US in the CS+US trials, consistent with it also playing a trace conditioning role of a CS in addition to any conditioning role of the auditory CS. Evidence of the sequential generalisation of this process may be seen in the appearance of a third v-CR prior to the implied third US, where the second US was also acting as trace CS.

## Summary and Conclusions

We have recorded non-invasively evoked and spontaneous activity from the human cerebellum during reflexive versus voluntary conditioning, the latter a form of Ivanov-Smolensky conditioning, making use of a vestibular double tap US. Despite weak classical conditioning with this US, we observed robust v-CRs, both to the auditory delay CS and the vestibular trace CS. In addition to the strong US-related changes in the ECeG, we also observed CR-related changes in the ECeG consistent with a role of the cerebellum in voluntary motor learning. The latter was accompanied by a CNV, indicative of a central mechanism in addition to any role of the cerebellum. The changes in the voluntary CRs from the first to second US tap are a form of sensorimotor adaptation as trace conditioning where each US can also double up as a CS. The generalisability of this should though be tested in a future experiment with longer sequences.

## Data Availability

Data is available on reasonable request.
